# Results of Vertebral Augmentation Treatment for Patients of Painful Osteoporotic Vertebral Compression Fractures: A Meta-Analysis of Eight Randomized Controlled Trials

**DOI:** 10.1371/journal.pone.0138126

**Published:** 2015-09-17

**Authors:** Lianhua Li, Jixin Ren, Jia Liu, Hao Wang, Xiaowei Wang, Zhi Liu, Tiansheng Sun

**Affiliations:** PLA Institute of Orthopedics, Beijing Army General Hospital, Beijing, 100700, China; University of Michigan, UNITED STATES

## Abstract

**Background:**

In 2009 two RCTs were publicated to question the efficacy of vertebroplasty comparing with sham treatment (ST) in the New England Journal of Medicine (NEJM), which provoked an academic debate on the efficacy of PVA. The purposes of our study were to compare clinical differences in pain relief, spinal functional outcomes, and overall quality of life between PVA and CT for painful osteoporotic VCFs.

**Methods:**

We searched PubMed, Cochrane Database of Systematic Reviews, Cochrane Central Register of Controlled Trials and Web of Knowledge from January 1980 to June 2013 with Medical Subject Headings terms and keywords. Risk of bias in the included studies was assessed in accordance with the Cochrane risk of bias tool. In this Meta-analysis dichotomous and continuous variables were calculated using the risk ratio (*RR*) and standardized mean difference (SMD), respectively.

**Results:**

Eight studies involving 987 patients met the criteria for inclusion. The VAS SMD was favoring the experimental group significantly (p < 0.001). Subgroup analysis suggested that the patients performed PVA with mean fracture age less than 3 months would got pain relief earlier and more durable than the control group (P <0.05). The SMD of spinal function assessed with RDQ and Oswestry LBP data was in favor of the experimental groups. QOL outcome improvement was demonstrated statistically significant at early, middle and late-term follow-up for PVA than the control group (P <0.05).

**Conclusions:**

In conclusion, this meta-analysis, which evaluated PVA for osteoporotic VCFs, demonstrated significant improvement regarding VAS, spinal function and QOL outcomes. The optimal fracture age was less than 12 weeks.

## Introduction

Since the development of percutaneous vertebral augmentation (PVA) for painful osteoporosis vertebral compression fractures (VCFs), there has been a rapid rise in the use of vertebroplasty (VP) and balloon kyphoplasty (BK) in the World. From 1993 to 2004 PVA had increased 12,900% in the number of procedures performed [1]. Furthermore, a consensus statement was developed to commend that PVA was safe and effective for treatment of osteoporotic VCFs by several American neurologic surgical and radiologic societies in 2009 [2]. In the same year two blinded placebo-controlled trials were publicated to question the efficacy of vertebroplasty comparing with sham treatment (ST) in the New England Journal of Medicine [3, 4], which provoked an academic debate on the efficacy of PVA. These publications had influenced the number of vertebroplasty referrals by decreasing nearly 50% at the Mayo Clinic [5]. American Academy of Orthopedic Surgeons clinical practice guideline based on an evidence-based approach recommended against vertebroplasty for osteoporotic VCFs patients in 2011 [6].

Nevertheless this was not the end of this story. After the 2 RCTs were publicated in the New England Journal of Medicine over 300 articles had been published annually on PVA, of which numerous commentaries and editorials had questioned the 2 trials in inclusion criterion, patients enrolling, revised power analysis, pain mechanism, high crossover, polymethylmeth-acrylate (PMMA) volume, et al [7–12]. Several other RCTs [13–18] have been published subsequently with contrary conclusions comparing VP or BK with conservative treatment (CT) in painful osteoporotic VCFs patients.

To address this controversy, we therefore performed this systematic review and meta-analysis with pooling available RCTs and differentiating the evidence based on differing controls, comparing the efficacy and safety of PVA with the control treatment for treatment of painful osteoporotic VCFs. The purposes of our study were to compare clinical differences in pain relief, spinal functional outcomes, and overall quality of life between PVA and CT for painful osteoporotic VCFs. Our aim was to obtain more comprehensive information that could help surgeons offer individualized clinical treatment to their patients.

## Methods

### Search strategy

Our search strategy followed the Preferred Reporting Items for Systematic Reviews and Meta-Analyses guidelines [19]. We searched PubMed, Cochrane Database of Systematic Reviews, Cochrane Central Register of Controlled Trials, Web of Knowledge, Chinese Biomedical Literature Database, and Wanfang Data. The electronic databases were searched from January 1980 to June 2013. The search strategy included Medical Subject Headings terms and keywords, such as the detailed search strategy for Pubmed was "((((((spinal compression fracture) OR vertebral compression fracture)) OR "Fractures, Compression"[Mesh])) AND (((((Vertebroplasty) OR kyphoplasty) OR vertebral augmentation)) OR (("Vertebroplasty"[Mesh]) OR "Kyphoplasty"[Mesh]))) AND (((random*) OR "Randomized Controlled Trials as Topic"[Mesh]) OR randomized controlled trial [Publication Type])". Reference lists of all the selected articles, previous reviews and meta-analyses were hand-searched for any additional articles. The search strategy was not limited by language.

### Eligibility criteria

Two review authors (Xiaowei Wang and Hao Wang) independently reviewed the abstracts and full text of articles to determine eligibility based on the criteria listed below. If a consensus could not be reached, a third review author (Lianhua Li) resolved the disagreement.

The following eligibility criteria were used when selecting the trials.

- Types of study: any published RCT or quasi-RCT that compared either VP or BK to CT, ST or other interventions for osteoporotic VCFs
- Types of participant: inclusion criteria: male and female patients aged >50 years who had an acute or chronic osteoporotic VCF that caused pain and functional limitations in their daily activities; exclusion criteria: patients with primary or secondary neoplasm, preexisting chronic pain or functional disability unrelated to vertebral fracture, and/or vertebral fractures without signal changes seen on magnetic resonance imaging
- Types of intervention: the experimental groups including VP or BK; the control groups including CT, ST or other interventions
- Type of outcomes: all studies including 1 or more of the following outcomes: Pain relief outcome: visual analogue scale (VAS); Spinal functional outcomes: Roland–Morris Disability Questionnaire (RDQ), Oswestry low back pain (LBP); Overall quality of life (QOL) outcomes: European Quality of Life–5 Dimensions (EQ-5D), Quality of Life Questionnaire of the European Foundation for Osteoporosis (QUALEFFO); Other outcomes: events of new VCF, new adjacent VCF and death

### Data extraction

Two review authors (Jia Liu and Jixin Ren) independently selected trials satisfying the inclusion criteria and extracted data for the outcomes using a data-extraction form. Relevant data included patients’ demographics, study characteristics, types of interventions, surgical procedures, and outcome parameters. Another review author (Lianhua Li) rechecked the extracted data. We used intention to treat data from trials wherever possible. If these were not available, we used data from the analysis of available data or data from the accompanying illustrations. If the data were not reported in the original article, we imputed them using appropriate methods, such as estimating the Mean and SD from the median(m), range(a,b)with the fomula:

*Mean* = (*a* + *b* + 2*m*)/4, *SD* = (*b* − *a*)/4, calculating the SD from 95%CI with the fomula:

$$SD=\frac{\sqrt{\text{N}\times \left(\text{upper limit}–\text{lower limit}\right)}}{3.92}$$

imputing the data equal to SD of the baseline SD for no other information available, et al.

### Assessment of methodological quality

The risk of bias in the included studies was independently assessed by two authors (Jia Liu and Hao Wang), in accordance with the Cochrane risk of bias tool [20]. It assessed factors such as sequence generation, allocation concealment, blinding of participants and personnel, blinding of outcome assessment, incomplete outcome data, selective outcome reporting and other issues. A third author (Lianhua Li) was the adjudicator when no consensus was achieved. We rated the risk of bias as low, unclear, or high according to established criteria.

### Statistical analysis

Meta-analyses were performed using STATA 12.0 software (Stata Corporation, College Station, TX, USA). For continuous variables, the mean difference from baseline to end point was calculated. We compared the experimental group to the control group by standardized mean difference (*SMD*) and 95% *CI*. For dichotomous outcomes, the risk ratio (*RR*) and 95% confidence interval (*CI*) were assessed. The studies were assumed to be heterogeneous and a random model was first applied. Then we used the Galbraith plot to assesse heterogeneity in this meta-analysis. If any points lied out the confidence bounds which illustrated heterogeneity, sensitivity analysis was performed to detect the resource of heterogeneity, and the meta-analysis was performed again with a fixed model after the corresponding RCT was omitted. Publication bias was assessed by visual inspection of funnel plots. If asymmetric, the trim and fill method was used to account for publication bias. We conducted planned subgroup analyses to reinvestigate pain relief by intervention methods (VP vs CT; VP vs ST; BK vs CT), mean fracture age (more than 3 months or less than 3 months), MRI as an inclusion criterion(yes or not). Apart from this, ITT analysis (yes or not) and crossover (yes or not) subgroup analysis were performed to examinate their affection to RCTs.

## Results

### Characteristics of eligible studies

After a complete systematic review was performed, 12 RCTs [3, 4, 13–18, 21–24] met the inclusion criteria (S1 Fig). Of the 12 RCTs, one RCT[17] had a later report [18] with intention-to-treat (ITT) analysis by the same authors, another RCT[21] had patients coming from one centre participanted in the previously published multi-centres FREE trial[24] and 2 RCT [14, 22] were update of one investigation [24] by the different authors. All these RCTs shared of the same group of patients were considerd as one RCT named by the latest one, leaving a total of 8 RCTs [3, 4, 13–16, 18, 22] for meta-analysis. Of the 8 RCTs, 5 RCTs [13, 15, 16, 18, 23] compared PV with CT, 2 RCTs [3, 16] compared PV with ST and 1 RCT [22] compared BK with CT. They consisted of 987 patients (759 males and 229 females), with the individual study sample size ranging from 34 to 300 patients. In all, the experimental group include 495 patients and the control group include 492 patients. The main characteristics of the 8 RCTs included in the meta-analysis were presented in S1 Table.

### Bias assessment

Of the 8 RCTs, all trials reported adequate sequence generation, 5 trials [3, 4, 15, 16, 18] reported adequate allocation concealment and the others gave insufficient information. All trials provided no blinding of participants and personnel during the study and we can not judge that whether the outcome was likely to be influenced by lack of blinding of participants and personnel. Three trials [3, 4, 15] reported blinding of outcome assessment. There were 4 trials [13, 16, 18, 22] that reported missing information such as loss to follow up and declining participation, of which missing data was balanced in numbers [18], imputed using appropriate methods [16], analyzed with a pattern mixture analysis [13] and ITT analysis [22]. For evaluating reporting bias due to selective outcome reporting, 3 trials [3, 4, 23] changed original study design and only one trial [23] was high risk bias, and the other trials’ power was sufficient to address the primary aim. In addition to this, baseline data was reported incompletely one trial [18] so that it can not be entered in meta-analysis. Three trials [4, 15, 23] had high crossover and one trial [22] was funded by the Medtronic Spine LLC, which could be potential sources of bias. The methodological quality of the included studies was illustrated in S2 Fig and presented as percentages in S3 Fig.

### VAS

Pain was assessed on VAS score of 0 to 10, with higher numbers indicating more pain. The VAS data was summarized as the VAS at the early-term follow-up (1 week-1 month), the middle-term follow-up (2–3 months) and the late-term follow-up (1 year).

I^2^ test for heterogeneity was performed and the results can be seen in S2 and S3 Tables. The VAS SMD was 0.30 (95%CI 0.09,0.51) for early, 0.28 (95%CI 0.14, 0.42) for middle and 0.26 (95%CI 0.12, 0.41) for late time points, favoring the experimental group, all of which were significant (p < 0.001) (see S2 Table, S4 Fig). After the Galbraith plot (S5 Fig) and sensitivity analysis (S6 Fig) were performed, 2 RCTs [13, 15] and one RCT[13] were omitted respectively at early-term and middle-term follow-up, and the fixed model meta-analysis was performed again which did not change the significant results (see S3 Table, p < 0.001). Publication bias was identified in the early-term follow-up because the funnel plot was asymmetric, but there was not significant in Egger’s test with the P value = 0.932 (S7 Fig). The trim and fill analysis (S8 Fig) showed that the adjusted SMD calculated under a random effects model was 0.49 (95%CI 0.25, 0.72), which revealed significant pain relief favoring the experimental group (P < 0.001).

S4 Table presented the results of subgroup analysis for VAS score. In the subgroup analysis, the efficacy of VP and BK on pain relief was greater than CT in all timepoint and the difference was significant except VP in early-term follow-up (P = 0.06), and there was no difference in pain scoring between VP and ST in the early- and middle-term follow-up. Effect sizes were decreased with time point going on (S9 Fig **)**. Another subgroup analysis suggested that the patients performed PVA with mean fracture age less than 3 months would got pain relief earlier and more durable than the control group (P <0.05). Subgroup analysis showed that the benefit of taking MRI as an inclusion criterion was significantly in all timepoint (P <0.05). Pain relief difference was greater in the RCTs with ITT and allowing crossover between two groups (P <0.01).

### Spinal functional outcomes

We selected RDQ and Oswestry LBP as the assessable index to spinal functional outcomes, which were extracted at the early-term follow-up (1 week-1 month), the middle-term follow-up (2–3 months) and the late-term follow-up (1 years). I^2^ test for heterogeneity was performed and the results can be seen in S2 and S3 Tables. The results were shown in S2 Table. Five RCTs [3, 4, 16, 22, 23] reported the RDQ scores and one RCT [15] provided the Oswestry LBP data at early-term time point, and the SMD was 0.32 (95%CI 0.10, 0.54), which was statistically significant in favor of the experimental groups (P = 0.004). The spinal function improvement was also confirmed at middle—and late-term follow-up for PVA than the control group by SMD 0.24 (95%CI 0.05, 0.42) and 0.26 (95% CI 0.14, 0.38) respectively (P <0.05). After the Galbraith plot and sensitivity analysis were performed, one RCT [15] was omitted at early-term and one RCT [3] at middle-term follow-up, and the fixed model meta-analysis was performed again which did not change the significant results (S3 Table, p < 0.001). The funnel plots were symmetric at middle- and late-term follow-up and asymmetric at early-term follow-up, which demonstrated publication bias at early-term follow-up analysis. The adjusted SMD calculated by the trim and fill analysis under a random effect model was 0.32 (95%CI 0.10, 0.54) favoring the PVA group (P = 0.004).

### QOL outcomes

QUALEFFO and EQ-5D were marked out to assess the QOL outcome improvement, which were extracted at the early-term (1 weeks-1 month), the middle-term (2–3 months) and the late-term follow-up (1 year). I^2^ test for heterogeneity was performed and the results can be seen in S2 and S3 Tables. The mean values of EQ-5D change from baseline data were multiplied by –1 to ensure that all the scales point in the same direction because QUALEFFO score decrease with QOL outcome getting better whilst EQ-5D score increase. In one trail [18] the baseline of EQ-5D data was not similar with the mean 0.36 in VP group and 0.08 in CT group because of baseline data missing, and we omitted this trial. The SMDs shown in S2 Table demonstrated statistically significant QOL outcomes improvement at early, middle and late-term follow-up for PVA than the control group (P <0.05). After the Galbraith plot and sensitivity analysis were performed, one RCT [13] was omitted at middle-term, and the fixed model meta-analysis was performed again which did not change the significant results (see S3 Table, p < 0.001). Sensitivity analysis did not result in any statistical changes to the results. The funnel plots were symmetric at all time points, which demonstrated no obvious publication bias.

### Other outcomes

Seven RCTs [3, 13, 15, 16, 18, 22, 23] reported events of new VCFs between 2 weeks and 2 years follow-up, among which 5 RCTs [13, 15, 18, 22, 23] provided information about adjacent VCFs. The pooled analysis showed there was no significant difference in the risk of new VCFs or adjacent VCFs between the experimental groups and the control groups, respectively with RR 1.09 (95%CI 0.61, 1.97, P = 0.77) and 1.80 (95%CI 0.61, 5.30, P = 0.28). No death was reported directly attributable to either the experimental groups or the control groups in any of the studies. Six RCTs [3, 13, 15–17, 22] provided information about the deaths unrelated to the vertebral fractures. The pooled analysis showed no significant differences between the two groups, with RR 0.94 (95%CI 0.55, 1.62, P = 0.83).

## Discussion

This is a further meta-analysis to evaluate the efficacy and safety of PVA for treatment of painful osteoporotic VCFs. In our meta-analysis, we find that PVA improved pain relief, spinal function and QOL more rapidly and notably than did control treatment, with significant differences sustained throughout one year follow-up. For most outcome measures, the differences between the two groups were reducing gradually at 1 year because the control group improved over time, probably as a result of fracture healing. Studies which included with a mean fracture age from onset of pain to treatment less than 12 weeks, or that used MRI edema as a including criterion, tended to have greater pain relief in favore of PVA. PVA was associated with no significant increase in the incidence of new VCFs or adjacent VCFs.

The continuous data standing for pain relief and function improvement was the main outcome analyzed in this meta-analysis, which was also mostly concerned by patients. For trials pooled in this study used differents scales to evaluate spinal function and QOL outcomes, the SMD was chosen as the summary statistics. All trials used changes from baseline (also called treatment difference) as the primary outcome, and we decided to use SMD based on changes from baseline to a single scale. The standard deviation of changes from baseline was not reported commonly and we think that imputing the standard deviation did not alter the conclusions of the meta-analysis, on the contrary imputing these missing data can equally provide accurate results [25, 26].

Our Meta-analysis showed that PVA could relieve pain and improve spinal function more rapidly and notably than control treatment for osteoporotic VCFs throughout one year follow-up. Belkoff et al. reported that pain reduction was attributable to the immobility and inhibition of micromovements of the fractured fragment and the cytotoxic effect of PMMA, which relieves pain by damaging the terminal nerve [27, 28]. But Togawa et al reported that PMMA did not create a definitive thermic effect on pain reduction [29]. The exact mechanism of pain remission after performing PVA remains unclear. Several large cohort and long-term follow-up research had confirmed this issue. Tanigawa et al. summarized 194 patients with 500 VCFs treated by VP and found that the mean VAS score of 7.6 changed to 2.3 at 1 month, 1.5 at 1 year, and 1.0 at 7 years [30]. Anselmetti et al. reported the clinic outcomes of 1,634 osteoporotic VCF patients with mean 25 months follow-up, in which the mean VAS score of 7.94 significantly reduced to 1.12 (P < 0.001) and the median ODI values of 82% decreased to 6% (P < 0.001) [31]. Taiwan' s National Health Insurance claims data of 9238 patients who had been discharged after hospitalization for a first-ever VCF between 2004 and 2007 showed that patients receiving PVA had a consistently lower incidence of 7-day re-hospitalization [32].

Increased mortality had been detected after elderly hip fracture during the first year, and may persist for several years after fracture [33]. Whether the phenomenon of increased mortality exists in patients with osteoporotic VCFs is still unknown. In a retrospective cohort study to compare survivorship after PVA of osteoporotic VCFs with CT, Patients undergoing cementation had a significant improvement in survival compared with controls, with a 7- to 10-fold decreased mortality risk during the first year after treatment, regardless of age, sex, comorbidities, or number of fractures. Survival advantage of cementation is found regard-less of sex, age, and number of fractures or comorbidities [34]. In this Meta-analysis we did not find the significant difference in mortality between the two groups.

There are several meta-analyses performed to compare results of vertebral augmentation treatment for patients of painful osteoporotic vertebral compression fractures with nonoperative treatment. In 2007, Taylor et al. [35] reported a meta-analysis including eight comparative studies and 35 case series which concluded that balloon kyphoplasty was more effective than medical management of osteoporotic vertebral compression fractures and as least as effective as vertebroplasty. In 2013, Anderson et al. [36] performed a meta-analysis including six RCTs from January 1980 to July 2011, which concluded that cement augmentation could get greater pain relief, functional recovery, and health-related quality of life than nonoperative or sham treatment. By contrast, the main advantage of our study is admitting the most comprehensive and the latest research, which including eight RCTs from January 1980 to June 2013. In addition, our study elaborated on how to deal with the problem of data missing.

The patient population and treatment were different among the papers by Buchbinder et al.[3], Kallmes et al., Klazen et al. and Van Meirhaeghe et al. that contribute the majority of patients to this report. The main difference in patient population performed with PVA was the mean fracture age among the four papers, which was more old in papers by Buchbinder et al.[3] (mean 8.7 weeks) and Kallmes et al.[4] (mean 19.5 weeks) than papers by Klazen et al.[16] (mean 4.2 weeks) and Van Meirhaeghe et al.[22] (mean 4.8 weeks), which mean that most of the participants performed with PVA were chronic fractures in the papers by Buchbinder et al.[3] and Kallemes et al.[4], and the majority fractures were acute in the papers by Klazen et al.[16] and Van Meirhaeghe et al.[22] The subgroup analysis in our study suggested that the patients performed PVA with mean fracture age less than 3 months got pain relief earlier and more durable, which may be one of the reasons why the results in the two NEJM papers by Buchbinder et al.[3] and Kallmes et al.[4] were different with other RCTs.[13, 15, 16, 18, 22, 23] In addition, the methods of PVA were different in the four papers, of which the vertebroplasty was performed in papers by Buchbinder et al.[3], Kallmes et al.[4] and Klazen et al.[16], and the kyphoplasty was performed by Van Meirhaeghe et al.[22] This difference may affect the accuracy of our results.

Our meta-analysis had several limitations such as confounding factors of the fracture time (chronic or acute fractures), the volume of cement and different procedures (vertebroplasty or kyphoplasty), which related to the heterogeneities in included RCTs. Heterogeneity may be classified into three broad categories: methodological, clinical and statistical forms [37]. The methodological heterogeneity was identified in S3 Fig. The clinical heterogeneity may be come from differences in inclusion-exclusion criteria (for example the fracture age), treatment methods of both groups (for example VP, BK,SM and CT), surgical technique (for example PMMA volume) volume and time endpoints. For example in papers by Buchbinder et al.[3] and Kallmes et al.[4],patient selection (64% and 70% of patients declined to participate in those trials respectively) raised major concerns selection bias, and the sham procedure did not have a control group without intervention. Limitations of the sources for the studies will inevitably translate to this meta-analysis. In this study we used random model to accommodate statistical heterogeneity, and drawed the Galbraith plot and performed sensitivity analysis to explore the resourse RCT of heterogeneity. We performed the fixed model Meta-analysis after omitting the resourse RCT of heterogeneity and the results were not changed, which confirmed that the results were credible. At last we applied funnel plots and the trim and fill analysis to account for publication bias and assured the results.

In conclusions, this meta-analysis, which evaluated PVA for osteoporotic VCFs, demonstrated significant improvement regarding VAS, spinal function and QOL outcomes. The optimal fracture age was less than 12 weeks. New fracture rates and death rate were similar in both groups patients. Well-designed and long-time follow-up RCTs are recommended for future work.

## Supporting Information

S1 Fig Flow chart summarizing the selection process of the trials. (TIF)

S2 Fig Methodological quality of the included studies showing the the risk-of-bias of each study in this meta-analysis. (TIF)

S3 Fig Risk of bias. Each risk-of-bias item is demonstrated as percentages across all of the included studies in this meta-analysis.
(TIF)

S4 Fig Forest plot and tabulated data illustrating the SMD in the VAS scores. (TIF)

S5 Fig Galbraith plot to assess heterogeneity in this meta-analysis. Two points lied out the confidence bounds which illustrated heterogeneity came from 2 RCTs.
(TIF)

S6 Fig Sensitivity analysis detected that the RCTs by Farrokhi et al and Blasco et al were the resources of heterogeneity. (TIF)

S7 Fig Funnel plot to assess publication bias for the VAS score at early-term follow-up. The asymmetrical funnel plot means publication bias existed in this meta-analysis
(TIF)

S8 Fig The trim and fill analysis model was shown with funnel plot, in which the quadratic plots were filled in random-effect Meta-analysis. (TIF)

S9 Fig The chart determinated the VAS SMD change with different term point. (TIF)

S1 Table Main characteristics of the identified studies pooled in the meta-analysis. (DOCX)

S2 Table Effect size (SMD, random Hedges’g) and confidence intervals according to study characteristics. (DOCX)

S3 Table Effect size (SMD, fixed Hedges’g) and confidence intervals according to study characteristics. (DOCX)

S4 Table Subgroup analyses of the included studies at different times by different influential factors. (DOCX)

S1 Materials PRISMA 2009 Checklist. (DOC)
