# Supplementary figures and images for: Results of Vertebral Augmentation Treatment for Patients of Painful Osteoporotic Vertebral Compression Fractures: A Meta-Analysis of Eight Randomized Controlled Trials
Source: PLoS One. 2015 Sep 17;10(9):e0138126. doi: 10.1371/journal.pone.0138126 (PMC4574925; doi:10.1371/journal.pone.0138126)

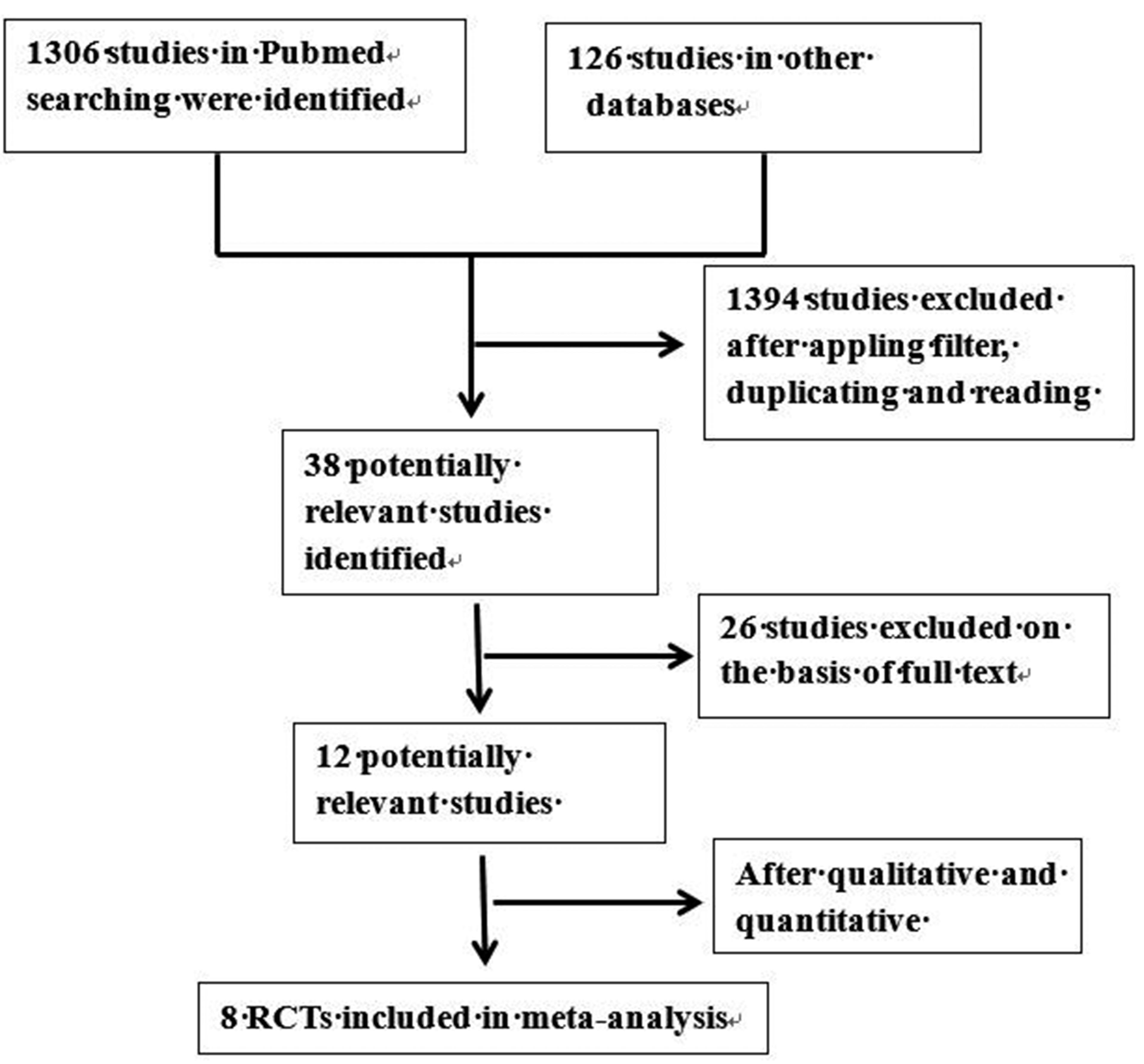

Supplement: S1 Fig — (TIF) [file pone.0138126.s001.tif]

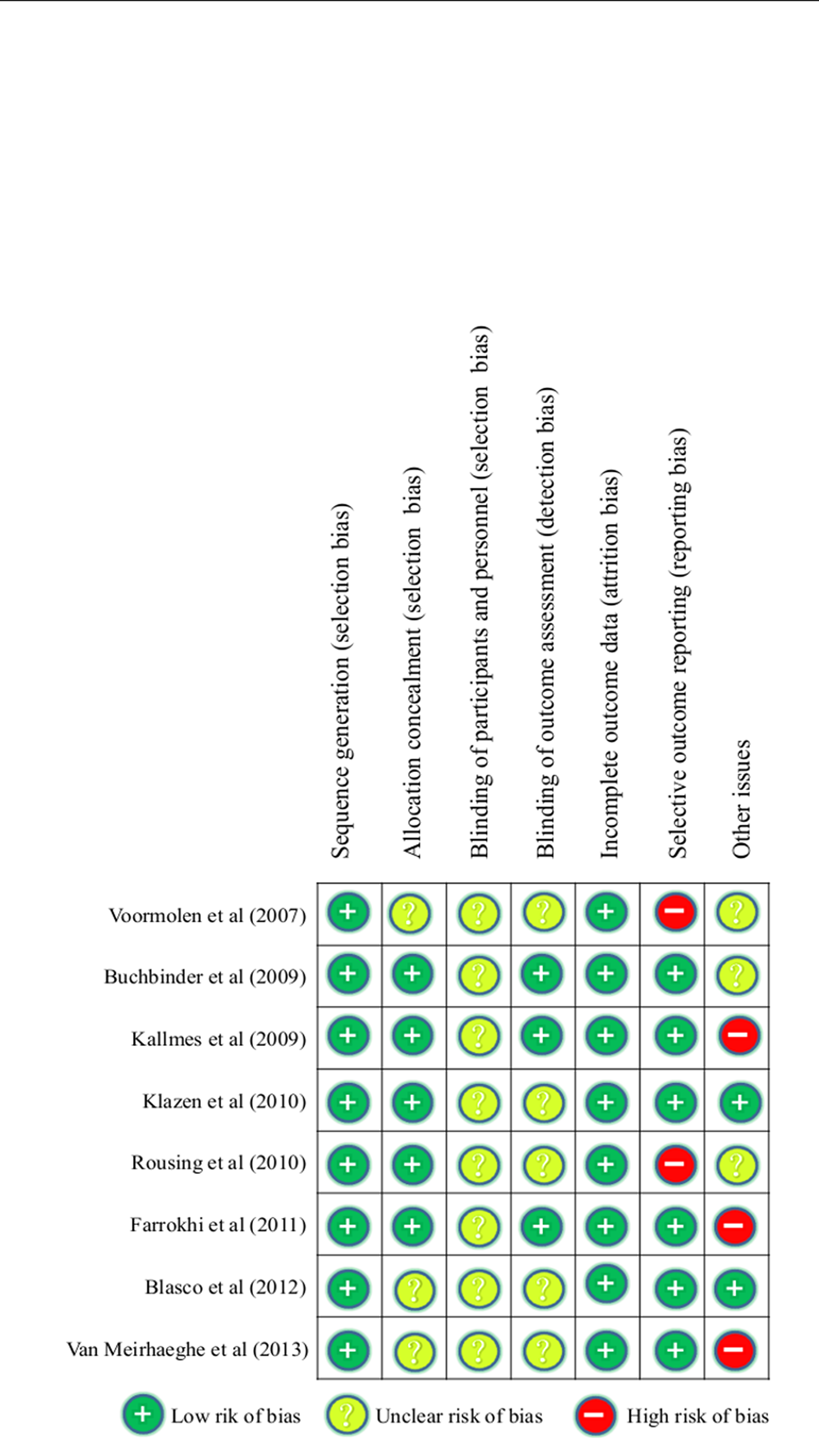

Supplement: S2 Fig — (TIF) [file pone.0138126.s002.tif]

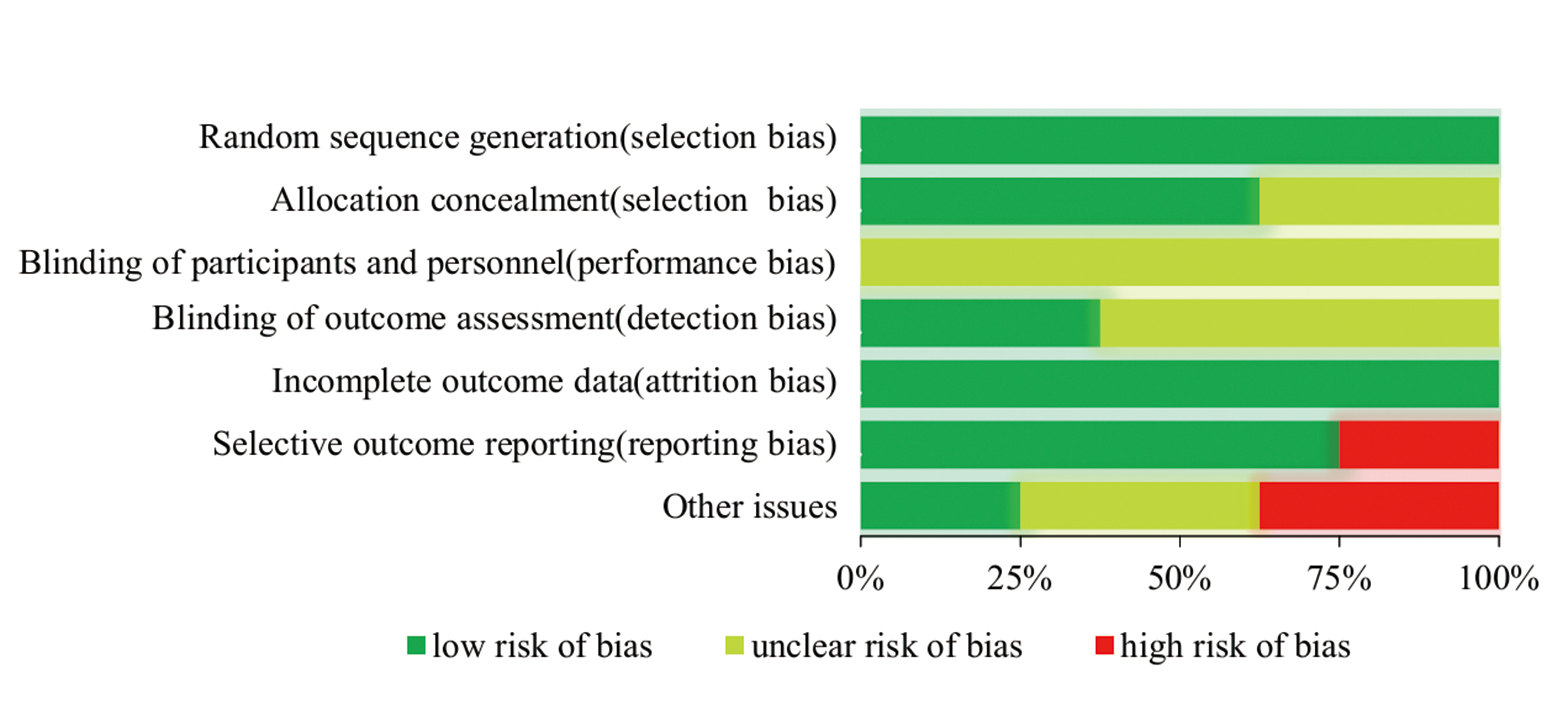

Supplement: S3 Fig — Each risk-of-bias item is demonstrated as percentages across all of the included studies in this meta-analysis. (TIF) [file pone.0138126.s003.tif]

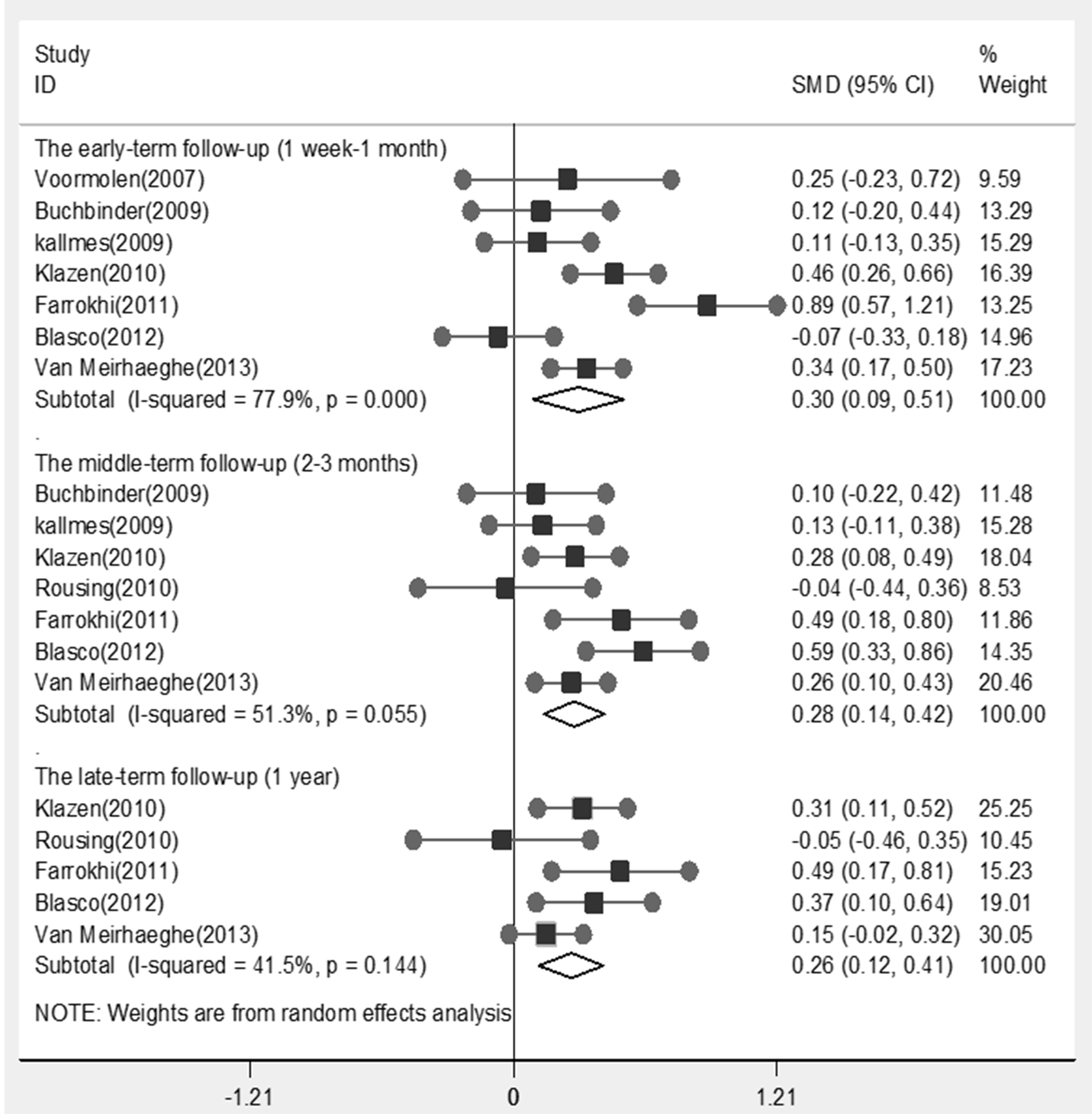

Supplement: S4 Fig — (TIF) [file pone.0138126.s004.tif]

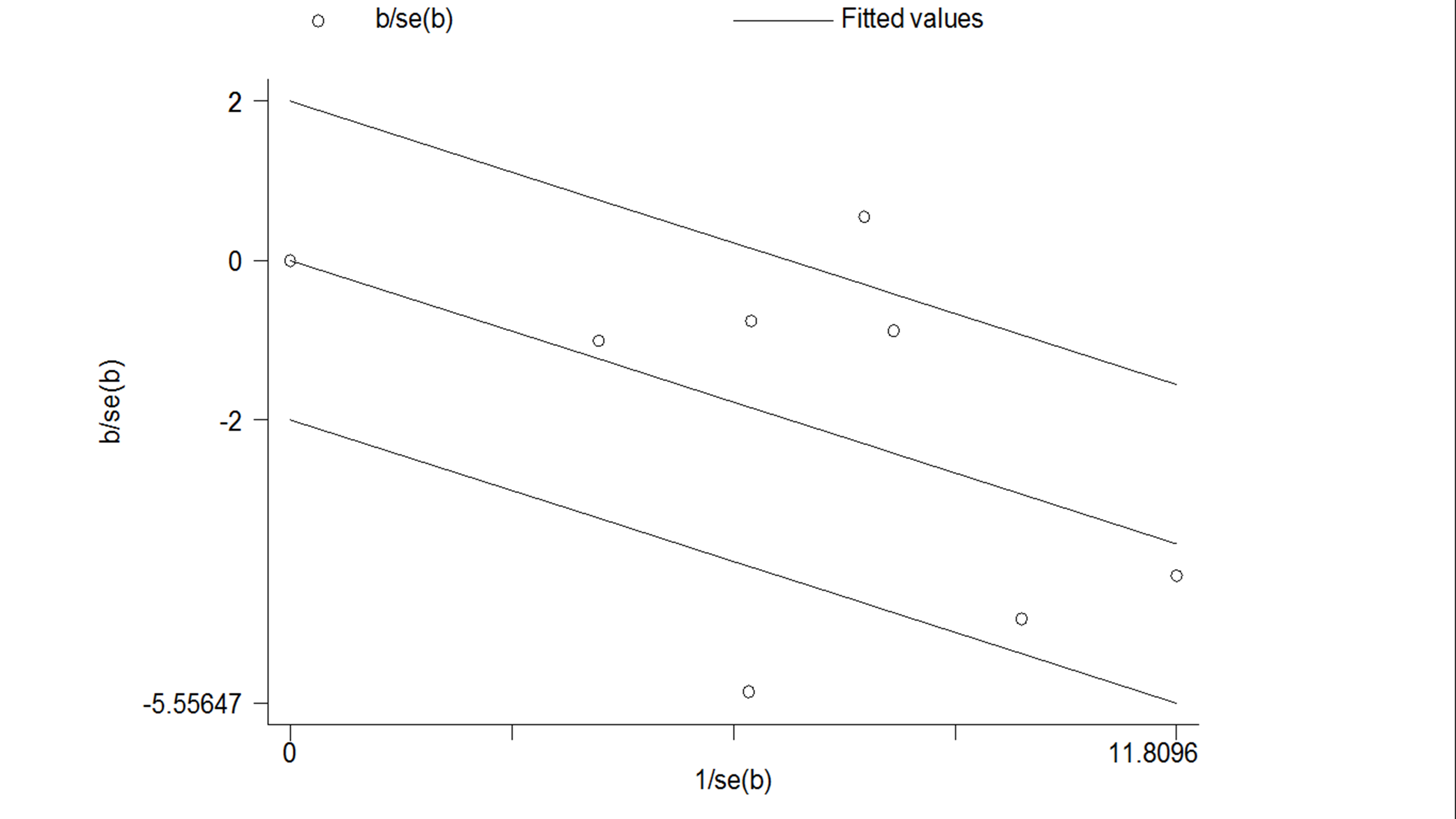

Supplement: S5 Fig — Two points lied out the confidence bounds which illustrated heterogeneity came from 2 RCTs. (TIF) [file pone.0138126.s005.tif]

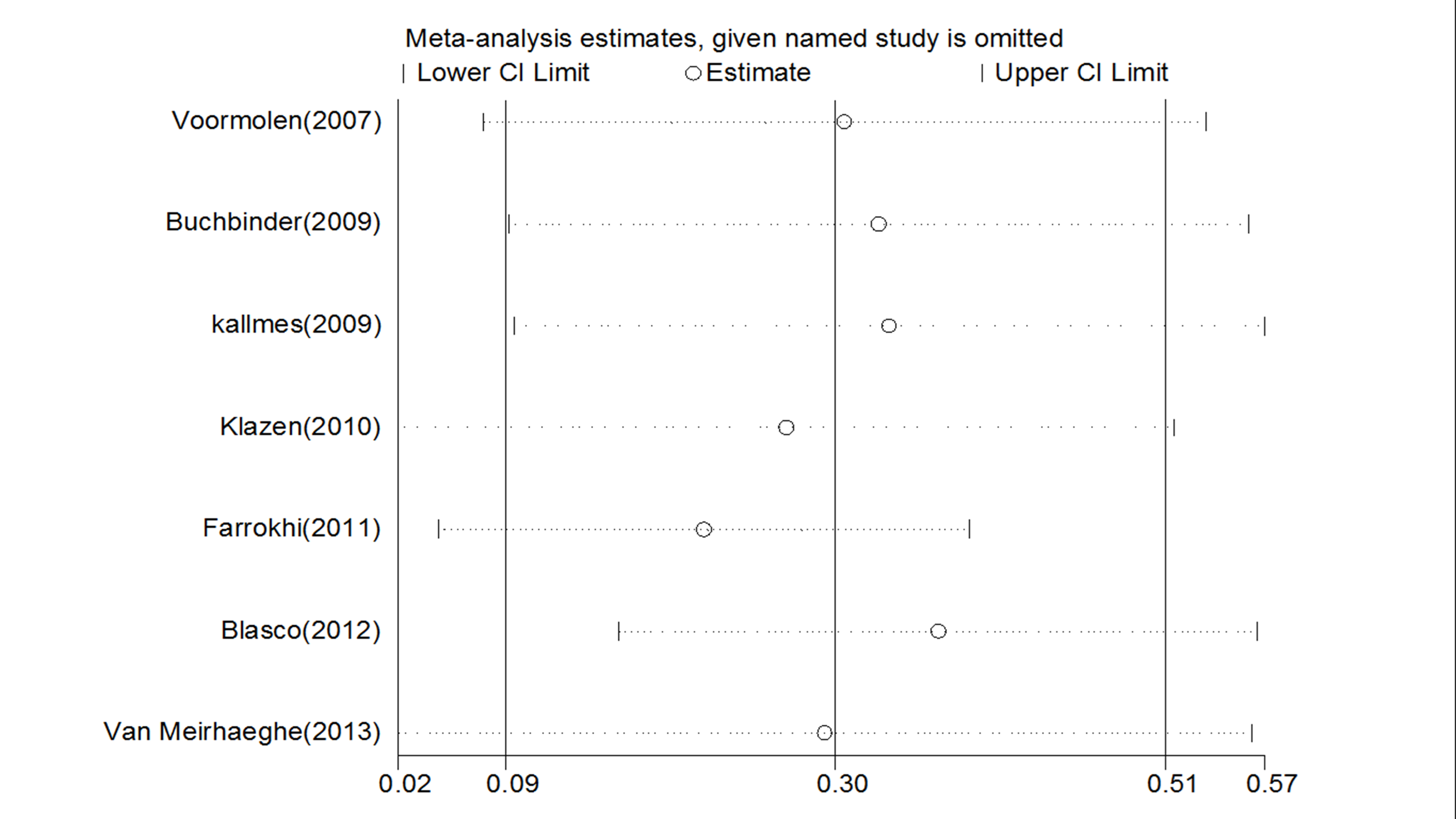

Supplement: S6 Fig — (TIF) [file pone.0138126.s006.tif]

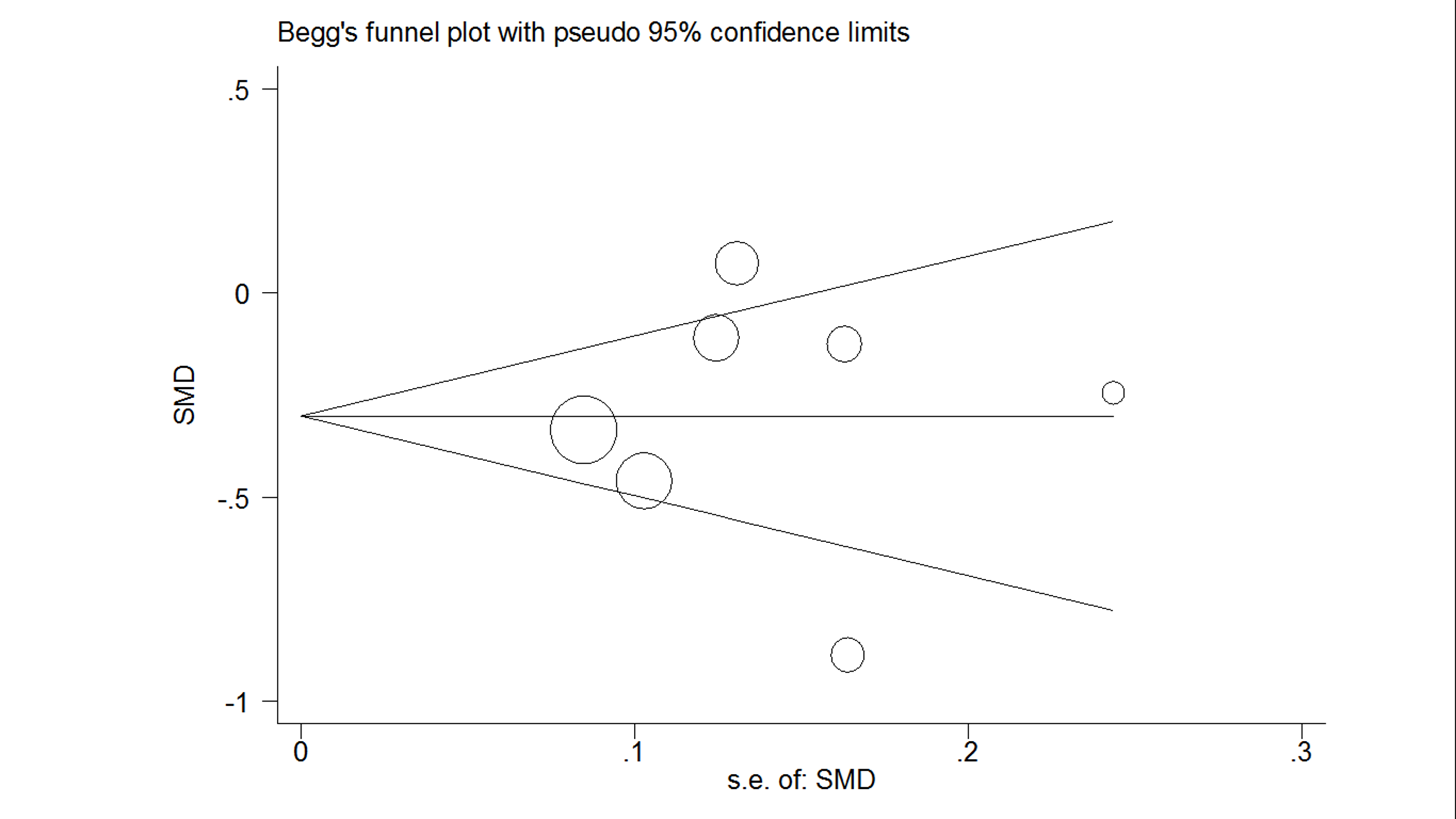

Supplement: S7 Fig — The asymmetrical funnel plot means publication bias existed in this meta-analysis (TIF) [file pone.0138126.s007.tif]

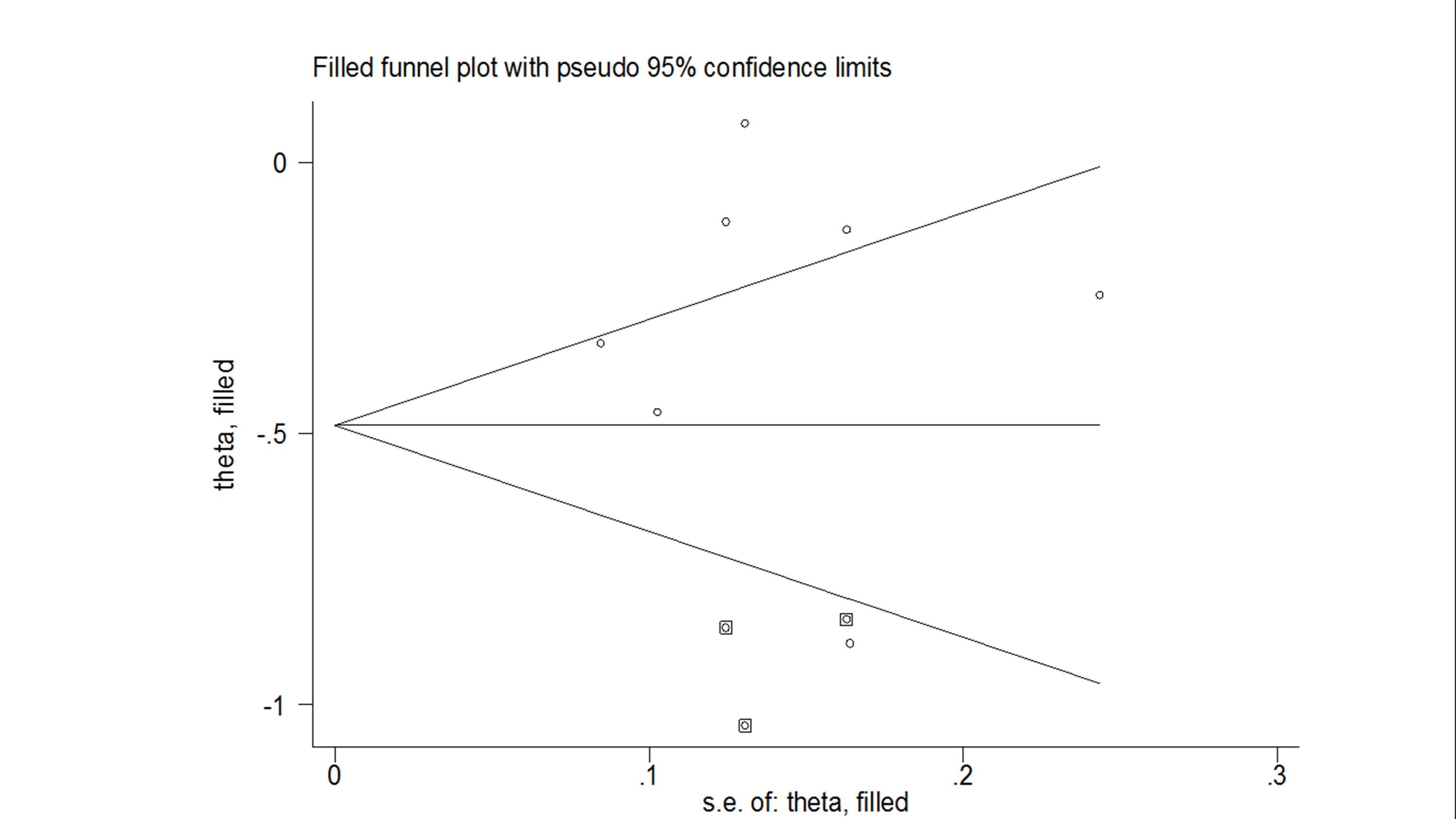

Supplement: S8 Fig — (TIF) [file pone.0138126.s008.tif]

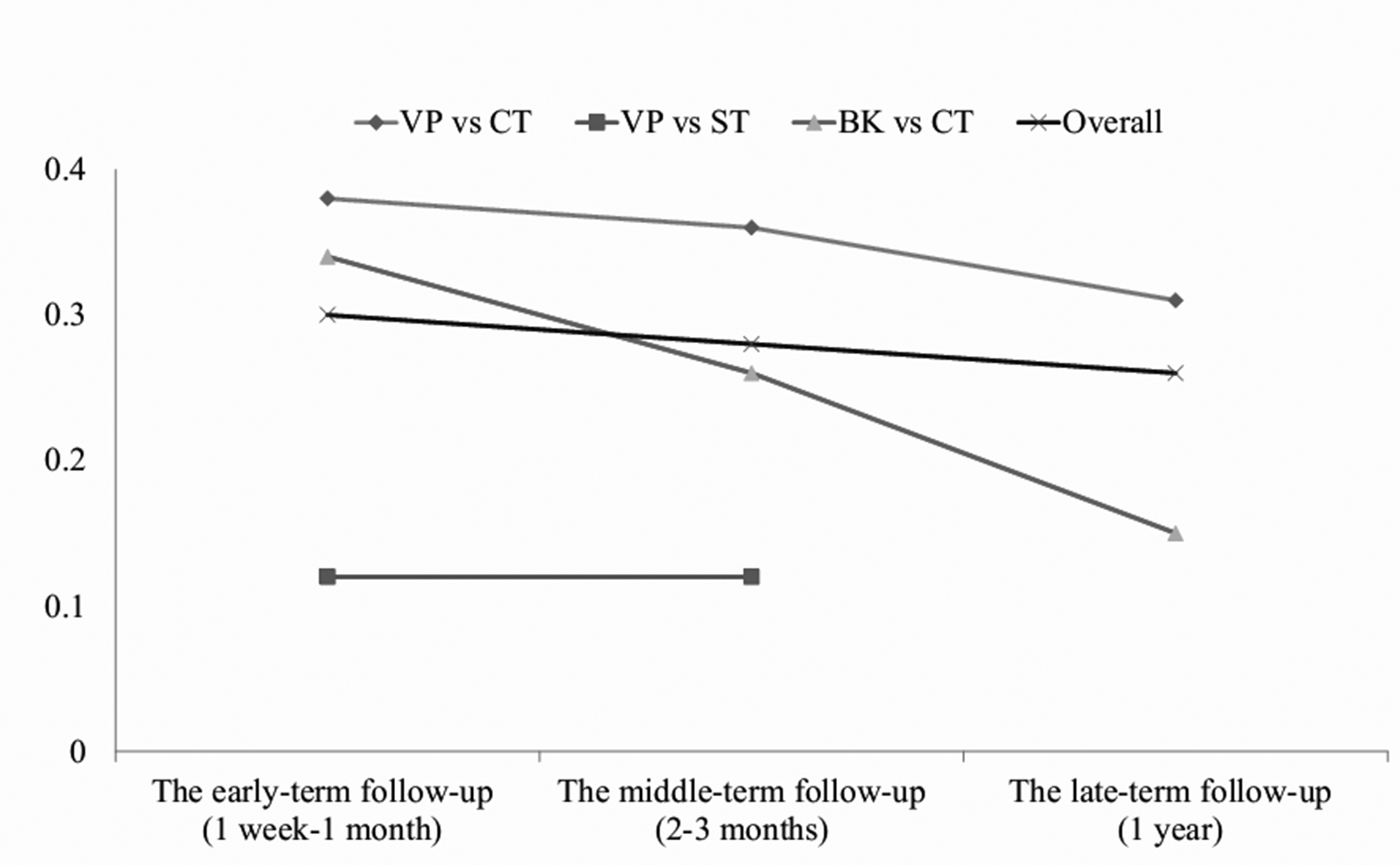

Supplement: S9 Fig — (TIF) [file pone.0138126.s009.tif]
