# Supplementary material for: Results of Vertebral Augmentation Treatment for Patients of Painful Osteoporotic Vertebral Compression Fractures: A Meta-Analysis of Eight Randomized Controlled Trials
Source: PLoS One. 2015 Sep 17;10(9):e0138126. doi: 10.1371/journal.pone.0138126 (PMC4574925; doi:10.1371/journal.pone.0138126)
Supplement: S1 Table — (DOCX) [file pone.0138126.s010.docx]

**S1 Table.** Main characteristics of the identified studies pooled in the meta-analysis

| Study | N  (EG/CG) | Multicentre  (number) | Intervention(EG/CG) | Duration  Analysed(months) | Sex(F/M) | Mean age(years) | Mean Fracture age(EG/CG, weeks) | Main Outcomes | Crossover to PVA (rate) |
| --- | --- | --- | --- | --- | --- | --- | --- | --- | --- |
| Voormolen (2007) | 18/16 | N | VP/CT | 0.5 | 28/6 | 73 | 12.7/12.1 | VAS;QUALEFFO;RDQ | Y(87.5%) |
| Buchbinder (2009) | 38/40 | Y(4) | VP/ST | 6 | 62/16 | 77 | 8.7/5.8 | VAS;QUALEFFO;RDQ;EQ-5D | N |
| kallmes (2009) | 68/63 | Y(11) | VP/ST | 3 | 99/32 | 74 | 19.5/21.5 | VAS;RDQ; SF-36; EQ-5D | Y(43%) |
| Klazen (2010) | 93/95 | Y(6) | VP/CT | 24 | 140/48 | 75 | 4.2/3.8 | VAS;EQ-5D;QUALEFFO;RDQ | Y(10%) |
| Rousing (2010) | 25/24 | N | VP/CT | 12 | 40/9 | 80 | 1.2/1.0 | VAS;SF-36;DPQ;EQ-5D;MMSE | N |
| Farrokhi (2011) | 40/42 | N | VP/CT | 36 | 60/22 | 73 | 27.0/30.0 | VAS;Oswestry LBP | Y(23.8%) |
| Blasco (2012) | 64/61 | N | VP/CT | 12 | 97/28 | 73 | 20.0/20.4 | VAS; QUALEFFO | N |
| Van Meirhaeghe(2013) | 149/151 | Y(21) | BK/CT | 24 | 232/68 | 73 | 4.8/5.3 | RDQ; SF-36; EQ-5D | N |

EG = the experimental groups; CG = the control groups; F = famale; M = male; Y = yes; N = no; VP = vertebroplasty; BK = balloon kyphoplasty; CT = conservative treatment; ST = sham treatment; QUALEFFO = Quality of Life Questionnaire of the European Foundation for Osteoporosis; RDQ = Roland-Morris Disability Questionnaire; DPQ = Dallas Pain Questionnaire; EQ-5D = European Quality of Life–5 Dimensions; MMSE = modified mini-mental state examination; SF-36= Short Form-36; LBP = low-back pain.

*All eight studies were randomized controlled trials.
