# Supplementary material for: Results of Vertebral Augmentation Treatment for Patients of Painful Osteoporotic Vertebral Compression Fractures: A Meta-Analysis of Eight Randomized Controlled Trials
Source: PLoS One. 2015 Sep 17;10(9):e0138126. doi: 10.1371/journal.pone.0138126 (PMC4574925; doi:10.1371/journal.pone.0138126)
Supplement: S2 Table — (DOCX) [file pone.0138126.s011.docx]

**S2 Table** Effect size (SMD, random Hedges’g) and confidence intervals according to study characteristics

| Outcomes | No.of RCTs | SMD(95%CI) | P _Heterogeneity_ | I^2^ | P_Z test_ |
| --- | --- | --- | --- | --- | --- |
| VAS | | | | | |
| The early term | 7 | 0.30 (0.09,0.51) | 0.000 | 77.9% | 0.005 |
| The middle-term | 7 | 0.28 (0.14,0.42) | 0.055 | 51.3% | 0.000 |
| The late-term | 5 | 0.26 (0.12,0.41) | 0.144 | 41.5% | 0.000 |
| Spinal function | | | | | |
| The early term | 6 | 0.32 (0.10, 0.54) | 0.001 | 75.7% | 0.004 |
| The middle-term | 5 | 0.24 (0.05, 0.42) | 0.022 | 65.2% | 0.011 |
| The late-term | 3 | 0.26 (0.14, 0.38) | 0.650 | 0.0% | 0.000 |
| QOL | | | | | |
| The early term | 6 | 0.23 (0.14,0.33) | 0.466 | 0.0% | 0.000 |
| The middle-term | 4 | 0.23 (0.05,0.41) | 0.056 | 60.3% | 0.012 |
| The late-term | 3 | 0.23 (0.11,0.34) | 0.366 | 0.6% | 0.000 |
