# Supplementary material for: Results of Vertebral Augmentation Treatment for Patients of Painful Osteoporotic Vertebral Compression Fractures: A Meta-Analysis of Eight Randomized Controlled Trials
Source: PLoS One. 2015 Sep 17;10(9):e0138126. doi: 10.1371/journal.pone.0138126 (PMC4574925; doi:10.1371/journal.pone.0138126)
Supplement: S3 Table — (DOCX) [file pone.0138126.s012.docx]

**S3 Table** Effect size (SMD, fixed Hedges’g) and confidence intervals according to study characteristics

| Outcomes | No.of RCTs | SMD(95%CI) | P _Heterogeneity_ | I^2^ | P_Z test_ |
| --- | --- | --- | --- | --- | --- |
| VAS | | | | | |
| The early term | 5 | 0.30 (0.20,0.41) | 0.184 | 35.4% | 0.000 |
| The middle-term | 6 | 0.24 (0.14,0.34) | 0.303 | 17.2% | 0.000 |
| The late-term | 5 | 0.25 (0.15,0.36) | 0.144 | 41.5% | 0.000 |
| Spinal function | | | | | |
| The early term | 5 | 0.24 (0.14, 0.35) | 0.213 | 31.3% | 0.000 |
| The middle-term | 4 | 0.29(0.19, 0.40) | 0.106 | 50.9% | 0.000 |
| The late-term | 3 | 0.26 (0.14, 0.38) | 0.650 | 0.0% | 0.000 |
| QOL | | | | | |
| The early term | 6 | 0.23 (0.14,0.33) | 0.466 | 0.0% | 0.000 |
| The middle-term | 3 | 0.19 (0.07,0.30) | 0.145 | 48.1% | 0.002 |
| The late-term | 3 | 0.23 (0.11,0.34) | 0.366 | 0.6% | 0.000 |
