# Supplementary material for: Results of Vertebral Augmentation Treatment for Patients of Painful Osteoporotic Vertebral Compression Fractures: A Meta-Analysis of Eight Randomized Controlled Trials
Source: PLoS One. 2015 Sep 17;10(9):e0138126. doi: 10.1371/journal.pone.0138126 (PMC4574925; doi:10.1371/journal.pone.0138126)
Supplement: S4 Table — (DOCX) [file pone.0138126.s013.docx]

**S4 Table** Subgroup analyses of the included studies at different times by different influential factors

| Factors |  | The early-term follow-up  (1 week-1 month) | |  |  | The middle-term follow-up  (2-3 months) | |  |  | The late-term follow-up  (1 year) | |  |
| --- | --- | --- | --- | --- | --- | --- | --- | --- | --- | --- | --- | --- |
|  | Subgroup (n) | | SMD (95%CI) | | Subgroup (n) | | SMD (95%CI) | | Subgroup (n) | | SMD (95%CI) | |
| Intervention methods | VP vs CT (4) | | 0.38(-0.02,0.78) p = 0.06 | | VP vs CT (4) | | 0.36(0.12,0.59) p = 0.003 | | VP vs CT (4) | | 0.31(0.14,0.49)  p = 0.000 | |
|  | VP vs ST (2) | | 0.12(-0.08,0.31) p = 0.245 | | VP vs ST (2) | | 0.12(-0.07,0.32) p = 0.219 | | VP vs ST (0) | | ── | |
|  | BK vs CT (1) | | 0.34(0.17,0.50) p = 0.000 | | BK vs CT (1) | | 0.26(0.10,0.43)  p = 0.002 | | BK vs CT (1) | | 0.15(-0.02,0.32)  p = 0.084 | |
| Mean fracture age | ＜ 3 months(3) | | 0.34(0.19,0.50)  p = 0.000 | | ＜3 months(4) | | 0.23(0.11,0.34)  p = 0.000 | | ＜3 months(3) | | 0.19(0.02,0.35)  p = 0.027 | |
|  | ＞ 3months(4) | | 0.29(-0.13,0.71) p = 0.181 | | ＞3months(3) | | 0.49(0.11,0.69) p = 0.007 | | ＞3months(2) | | 0.42(0.22,0.62)  p = 0.000 | |
| MRI as an inclusion criterion | Yes(4) | | 0.23(0.01,0.45)  p = 0.044 | | Yes(4) | | 0.31(0.15, 0.48) p = 0.000 | | Yes(3) | | 0.25(0.12,0.39)  p = 0.000 | |
|  | No(3) | | 0.42(-0.11,0.94) p = 0.119 | | No(3) | | 0.21(-0.08, 0.49) p = 0.154 | | No(2) | | 0.23(-0.30,0.77) p = 0.389 | |
| ITT analysis | Yes(5) | | 0.38(0.15,0.60)  p = 0.001 | | Yes(5) | | 0.25(0.15,0.36) p = 0.000 | | Yes(3) | | 0.28(0.10,0.46) p = 0.002 | |
|  | No(2) | | 0.02(-0.26,0.30) p = 0.886 | | No(2) | | 0.30(-0.32,0.92) p = 0.346 | | No(2) | | 0.19(-0.22,0.60) p = 0.373 | |
| Crossover | Yes(4) | | 0.43(0.11,0.75)  p = 0.009 | | Yes(3) | | 0.28(0.10 0.46)  p = 0.002 | | Yes(2) | | 0.37(0.19, 0.54) p = 0.000 | |
|  | No(3) | | 0.14(-0.12,0.41) p = 0.283 | | No(4) | | 0.26(0.02,0.50)  p = 0.033 | | No(3) | | 0.18(-0.01, 0.38) p = 0.063 | |
